# Supplementary material for: SARS‐CoV‐2 spike spurs intestinal inflammation via VEGF production in enterocytes
Source: EMBO Mol Med. 2022 Apr 19;14(5):e14844. doi: 10.15252/emmm.202114844 (PMC9081906; doi:10.15252/emmm.202114844)
Supplement: Supplementary file 1 — Expanded View Figures PDF [file EMMM-14-e14844-s001.pdf]

Expanded View Figures

Figure EV1. VEGF level correlates with disease progression of COVID-19.

A Immunofluorescence analysis of ACE2 and SARS-CoV-2 spike protein in the intestinal tissues of COVID-19 patients. ACE2 staining in green, spike protein staining in red and nuclear staining in blue. Scale bars, 50 μm.
B The percentage of spike stained positive in different parts of the intestinal tissues from patients with COVID-19. Number of samples for each group as indicated.
C ELISA analysis of VEGF concentration in plasma of COVID-19 patients with (n = 11) or without (n = 8) GI symptoms.
D ELISA analysis of VEGF concentration in plasma of COVID-19 patients with (n = 8) or without (n = 11) disease progression.
E Temporal course of plasma VEGF at the early and late stage of COVID-19 infection by ELISA analysis. Data shown are the levels of plasma VEGF in patients with (n = 8) and without (n = 11) disease progression at the early stage (one to three days after laboratory-confirmed for COVID-19) and late stage (more than three days after laboratory-confirmed for COVID-19).
F mRNA levels of VEGF-B and VEGF-C in the intestinal tissues from COVID-19 patients (n = 5) or healthy controls (n = 5) by RNA-seq.
G mRNA levels of VEGFR1, VEGFR2, and VEGFR3 in the intestinal tissues from COVID-19 patients (n = 5) or healthy controls (n = 5) by RNA-seq.

Data information: All data are shown as mean ± SD. P values are determined by Student's t-test.

 © 2022 The Authors

16 of 15 *EMBO Molecular Medicine* 14: e14844 | 2022 © 2022 The Authors

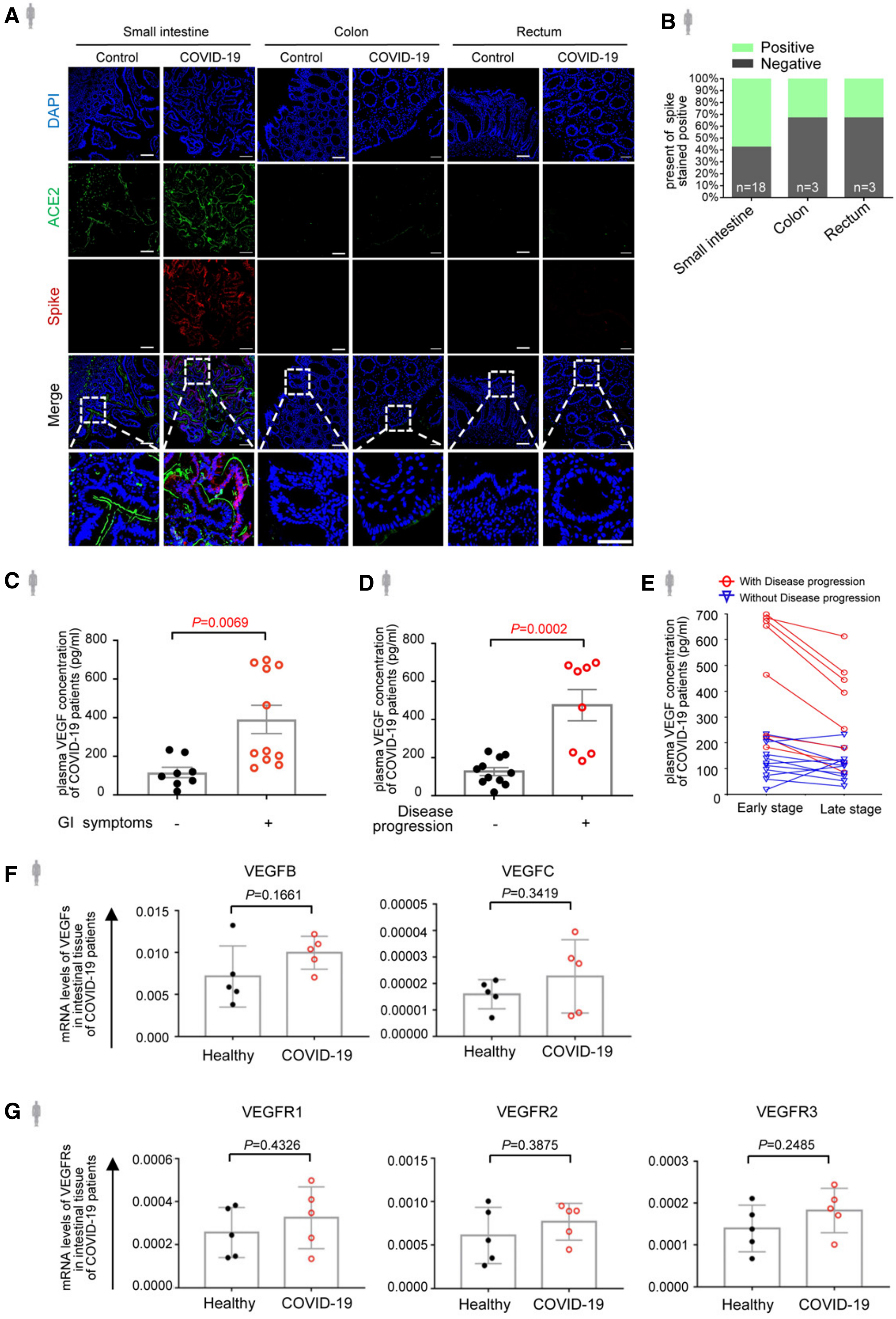
A
Small intestine
Colon
Rectum
Control
COVID-19
DAPI
ACE2
Spike
Merge
B
Positive
Negative
present of spike stained positive
n=18
n=3
n=3
Small intestine
Colon
Rectum
C
P=0.0069
plasma VEGF concentration of COVID-19 patients (pg/ml)
GI symptoms
D
P=0.0002
Disease progression
E
With Disease progression
Without Disease progression
Early stage
Late stage
F
VEGFB
P=0.1661
VEGFC
P=0.3419
mRNA levels of VEGFs in intestinal tissue of COVID-19 patients
Healthy
COVID-19
G
VEGFR1
P=0.4326
VEGFR2
P=0.3875
VEGFR3
P=0.2485
mRNA levels of VEGFRs in intestinal tissue of COVID-19 patients

Figure EV1.

Figure EV2. Generation of an animal model that mimics the intestinal inflammation in COVID-19.

A Recombinant SARS-CoV-2 spike consists of receptor binding domain (RBD, Arg319-Phe541) and Fc region at the C-terminus. Human IgG-Fc was adopted as control protein.

B Immunofluorescence analysis of ACE2 and spike RBD in murine intestinal tissue. ACE2 staining in green, spike RBD staining in red and nuclear staining in blue. Scale bar, 50 μm.

C, D The scatter chart shows the SARS-CoV-2 spike RBD staining (C) or the ACE2 staining (D) in murine intestinal tissues. Control-Fc, $n = 5$; Spike RBD-Fc, $n = 6$. n, biologically independent samples (mice).

E Representative H&E images of inflammation in the intestinal tissues from animals treated with only Spike RBD-Fc or Control-Fc. Scale bar, 100 μm. Control-Fc, $n = 1$; Spike RBD-Fc, $n = 1$. n, biologically independent samples (mice).

F qRT–PCR analysis of levels of inflammatory factors in the intestinal tissues of mice treated with Control-Fc or Spike RBD-Fc. Control-Fc, $n = 5$; Spike RBD-F, $n = 6$. n, biologically independent samples (mice).

Data information: All data are shown as mean ± SD. For (C) and (D), P values are determined by one-way ANOVA; for (F), P values are determined by Student's t-test.

 © 2022 The Authors

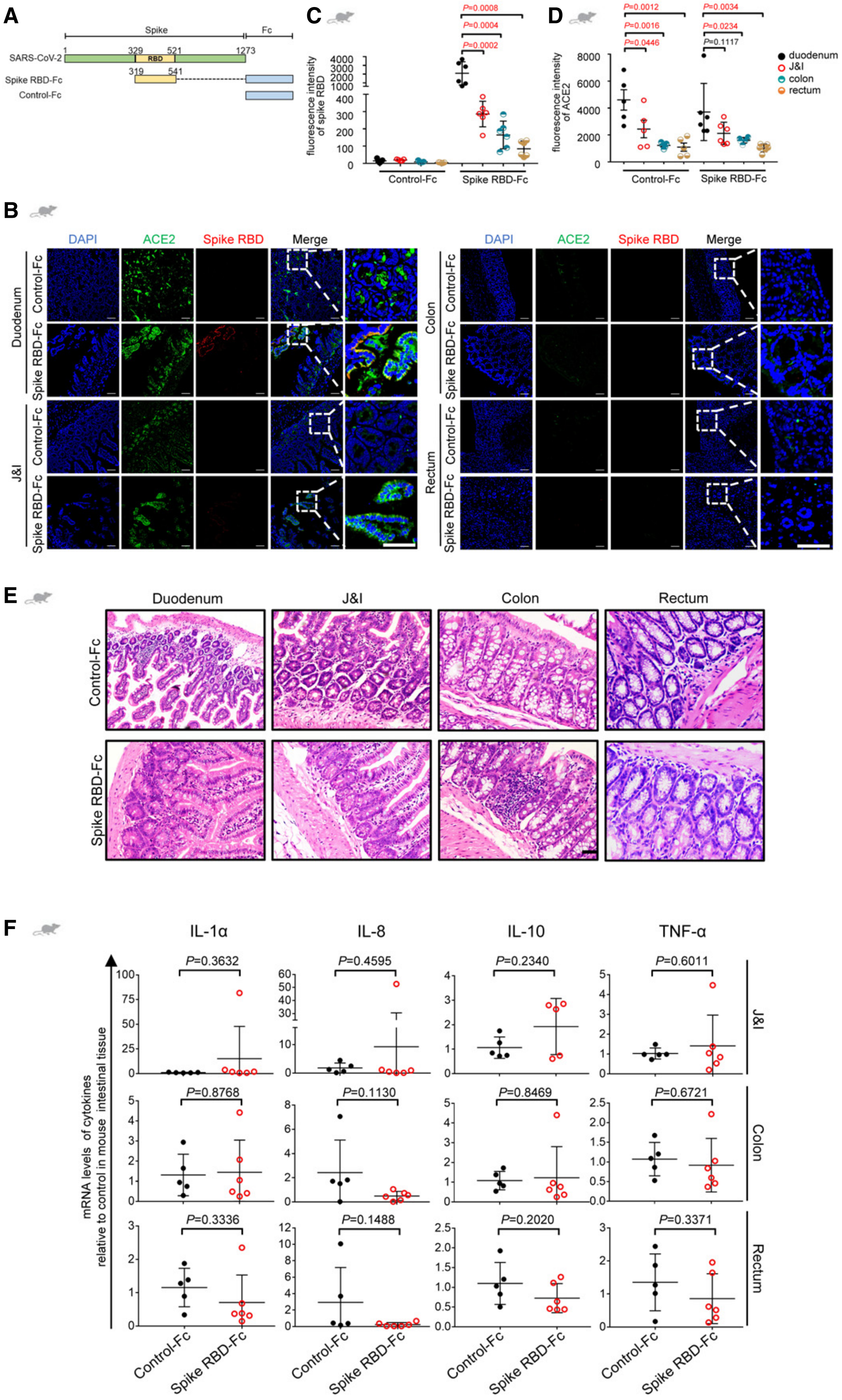
A
Spike
Fc
SARS-CoV-2
Spike RBD-Fc
Control-Fc
RBD
1
329
521
1273
319
541
C
fluorescence intensity of spike RBD
Control-Fc
Spike RBD-Fc
P=0.0008
P=0.0004
P=0.0002
D
fluorescence intensity of ACE2
Control-Fc
Spike RBD-Fc
P=0.0012
P=0.0016
P=0.0446
P=0.0034
P=0.0234
P=0.1117
duodenum
J&I
colon
rectum
B
DAPI
ACE2
Spike RBD
Merge
Duodenum
J&I
Colon
Rectum
Control-Fc
Spike RBD-Fc
E
Duodenum
J&I
Colon
Rectum
Control-Fc
Spike RBD-Fc
F
IL-1α
IL-8
IL-10
TNF-α
mRNA levels of cytokines relative to control in mouse intestinal tissue
P=0.3632
P=0.4595
P=0.2340
P=0.6011
P=0.8768
P=0.1130
P=0.8469
P=0.6721
P=0.3336
P=0.1488
P=0.2020
P=0.3371
J&I
Colon
Rectum
Control-Fc
Spike RBD-Fc

Figure EV2.

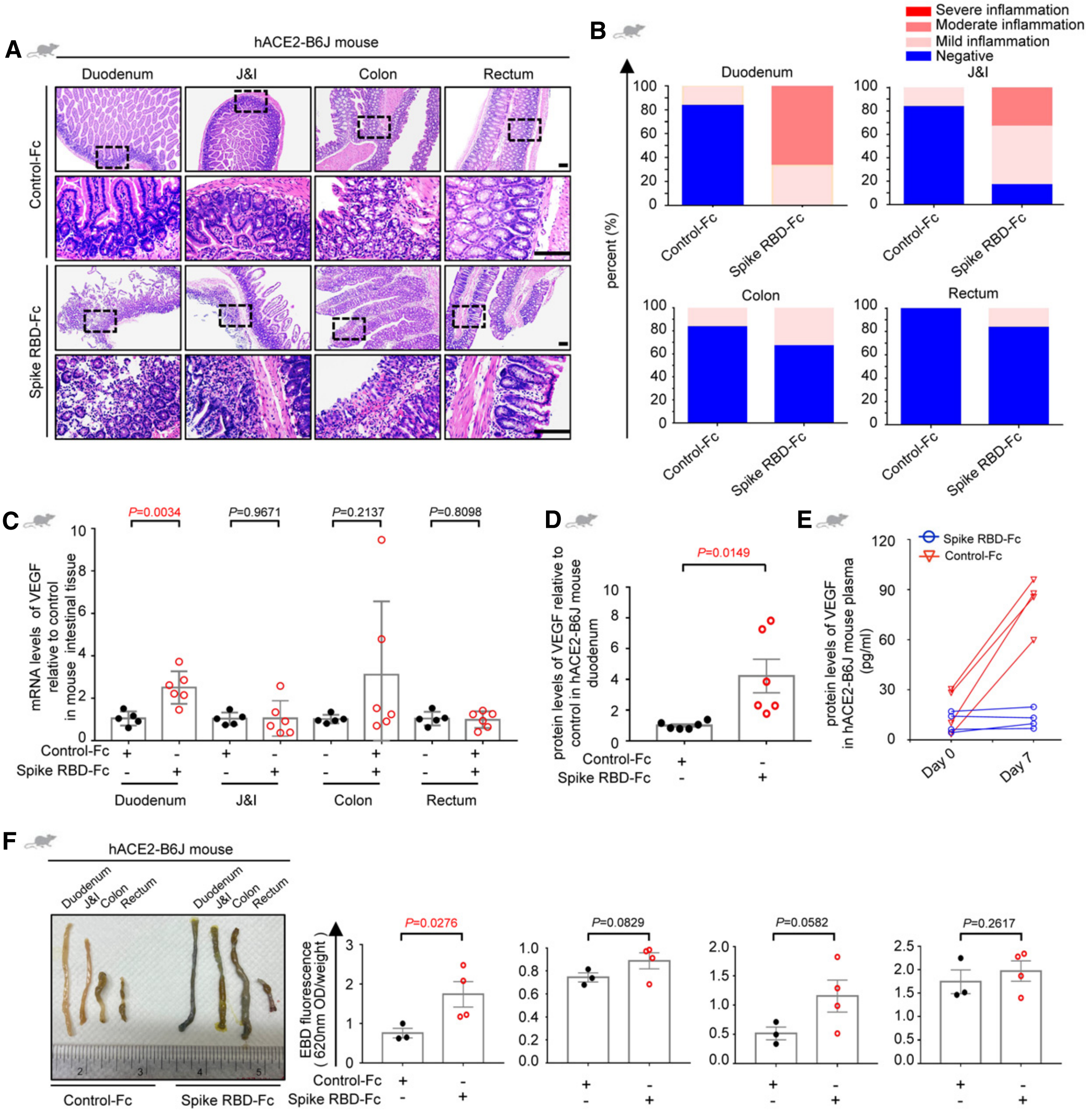

Figure EV3. SARS-CoV-2 spike protein induces intestinal permeability via VEGF overproduction.

A, B Representative H&E images (A) and the quantitative analysis of inflammation (B) of the intestinal tissues from hACE2-B6J mouse treated with Spike RBD-Fc or Control-Fc. Scale bar, 100 μm. For each group, n = 6. n, biologically independent samples (mice).

C qRT–PCR analysis of levels of VEGF in the intestinal tissues of mice treated with Control-Fc or Spike RBD-Fc. Control-Fc, n = 5; Spike RBD-Fc, n = 6. n, biologically independent samples (mice).

D ELISA analysis of VEGF concentration in duodenum tissue of hACE2-B6J mice treated with Control-Fc or Spike RBD-Fc. For each group, n = 6. n, biologically independent samples (mice).

E ELISA analysis of VEGF concentration in plasma of hACE2-B6J mice after six hours treatment with Control-Fc or Spike RBD-Fc, or seven days after treated with Control-Fc or Spike RBD-Fc daily. For each group, n = 4. n, biologically independent samples (mice).

F Evaluation of intestinal permeability in hACE2 B6J mice after seven days treatment with Control-Fc or Spike RBD-Fc daily by the Evans Blue dye extravasation assay. Control-Fc, n = 3; Spike RBD-Fc, n = 4. n, biologically independent samples (mice).

Data information: All data are shown as mean ± SD. *P* values are determined by Student's *t*-test.

© 2022 The Authors

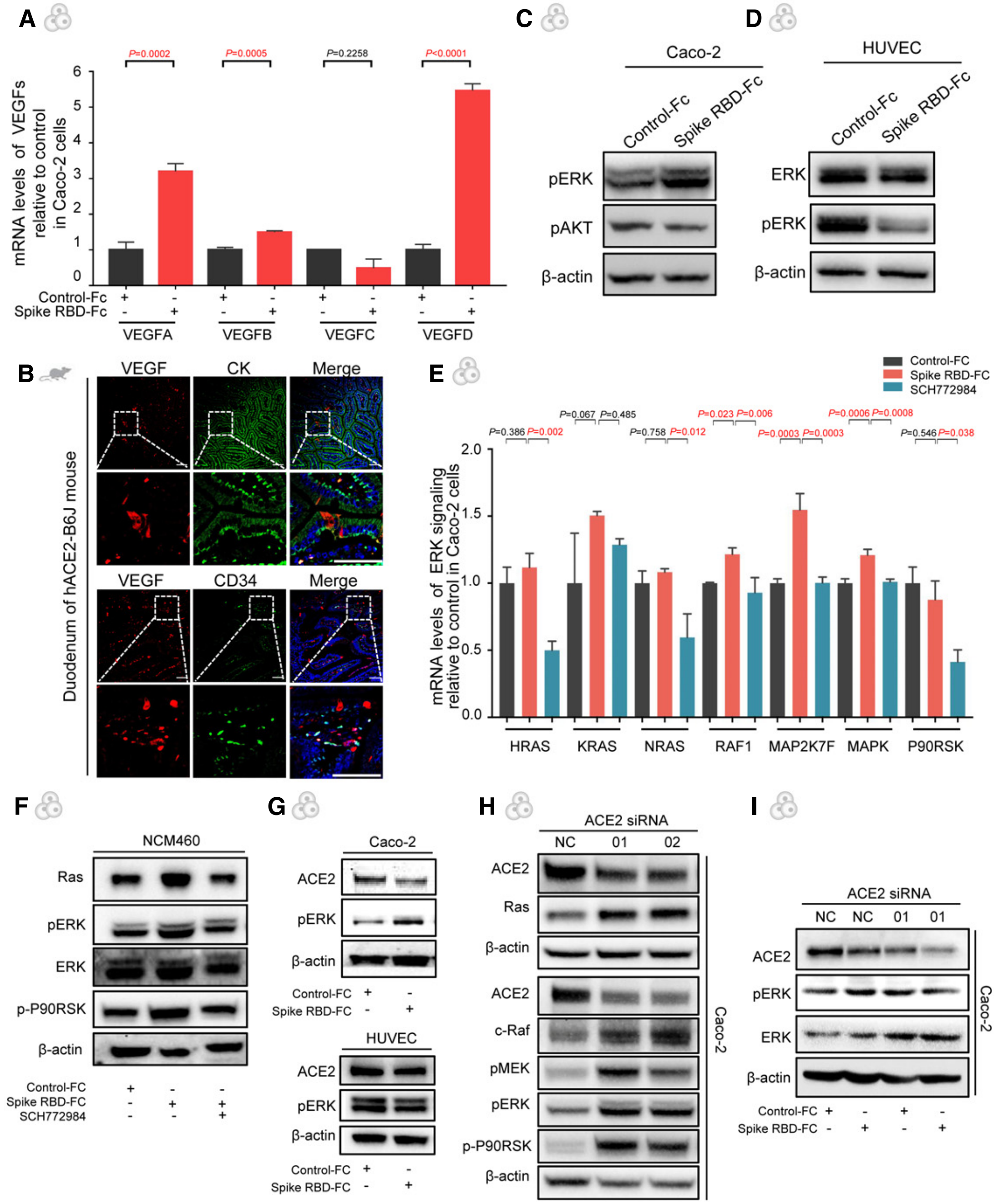
A
mRNA levels of VEGFs relative to control in Caco-2 cells
P=0.0002
P=0.0005
P=0.2258
P<0.0001
Control-Fc
Spike RBD-Fc
VEGFA
VEGFB
VEGFC
VEGFD
B
Duodenum of hACE2-B6J mouse
VEGF
CK
Merge
CD34
C
Caco-2
Control-Fc
Spike RBD-Fc
pERK
pAKT
β-actin
D
HUVEC
ERK
E
Control-FC
Spike RBD-FC
SCH772984
mRNA levels of ERK signaling relative to control in Caco-2 cells
P=0.386
P=0.002
P=0.067
P=0.485
P=0.758
P=0.012
P=0.023
P=0.006
P=0.0003
P=0.0006
P=0.0008
P=0.546
P=0.038
HRAS
KRAS
NRAS
RAF1
MAP2K7F
MAPK
P90RSK
F
NCM460
Ras
p-P90RSK
G
ACE2
H
ACE2 siRNA
NC
01
02
c-Raf
pMEK
Caco-2
I

Figure EV4.

Figure EV4. SARS-CoV-2 spike promotes VEGF production in enterocytes via the ERK pathway.

A qRT–PCR analysis of the transcription level of VEGF isoforms in Caco-2 cells treated with the Control-Fc or Spike RBD-Fc. Data are from three technical replicates with similar results from three biological replicates.
B Immunofluorescence analysis of VEGF, CK, and CD34 protein in the duodenum of hACE2-B6J mouse. CK and CD34 staining in green, VEGF staining in red and nuclear staining in blue. Scale bars, 50 μm. Representative images of two to three biological replicates.
C The expression of the pERK and pAKT in Caco-2 cells were detected by Western blot. Caco-2 cells were treated with Control-Fc or Spike RBD-Fc. β-actin was used as a reference gene.
D The expression of the ERK and pERK in HUVECs were detected by Western blot. HUVECs were treated with Control-Fc or Spike RBD-Fc. β-actin was used as a reference gene.
E qRT–PCR analysis of the transcription level of ERK pathway in Caco-2 cells treated with the Control-Fc or Spike RBD-Fc. Data are from three technical replicates with similar results from three biological replicate experiments.
F The levels of Ras, pERK, ERK, and p-P90RSK in NCM460 cells treated with Control-Fc, Spike RBD-Fc or Spike RBD-Fc combined with SCH772984 by Western blot. β-actin was used as a reference gene.
G The levels of ACE2 and pERK in Caco-2 and HUVEC cells treated with Control-Fc or Spike RBD-Fc by Western blot. β-actin was used as a reference gene.
H The levels of Ras, c-Raf, pMEK, pERK, ERK, and p-P90RSK in Caco-2 with ACE2 knockdown by Western blot. β-actin was used as a reference gene.
I The levels of ACE2, pERK, and ERK in Caco-2 cells treated with Control-Fc, Spike RBD-Fc with ACE2 knockdown by Western blot. β-actin was used as a reference gene.

Data information: All data are shown as mean ± SD. For (A), *P* values are determined by Student's *t*-test; for (E), *P* values are determined by one-way ANOVA.

 © 2022 The Authors

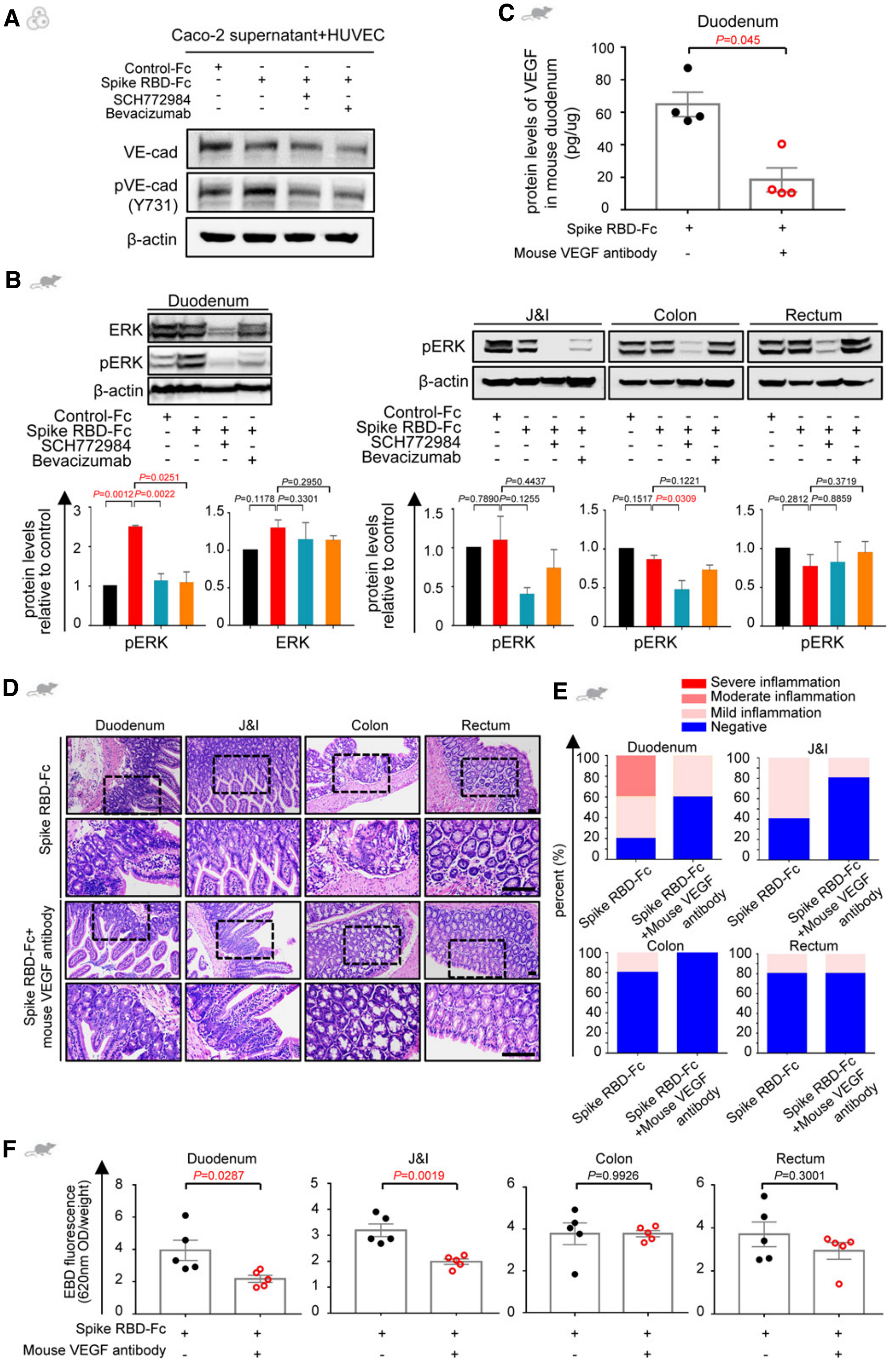

A
Caco-2 supernatant+HUVEC
Control-Fc
Spike RBD-Fc
SCH772984
Bevacizumab
VE-cad
pVE-cad (Y731)
β-actin
C
Duodenum
P=0.045
protein levels of VEGF in mouse duodenum (pg/ug)
Spike RBD-Fc
Mouse VEGF antibody
B
Duodenum
ERK
pERK
β-actin
J&I
Colon
Rectum
pERK
β-actin
Control-Fc
Spike RBD-Fc
SCH772984
Bevacizumab
P=0.0251
P=0.0012
P=0.0022
P=0.2950
P=0.1178
P=0.3301
P=0.4437
P=0.7890
P=0.1255
P=0.1221
P=0.1517
P=0.0309
P=0.3719
P=0.2812
P=0.8859
protein levels relative to control
pERK
ERK
D
Duodenum
J&I
Colon
Rectum
Spike RBD-Fc
Spike RBD-Fc+ mouse VEGF antibody
E
Severe inflammation
Moderate inflammation
Mild inflammation
Negative
percent (%)
Spike RBD-Fc
Spike RBD-Fc +Mouse VEGF antibody
F
Duodenum
P=0.0287
J&I
P=0.0019
Colon
P=0.9926
Rectum
P=0.3001
EBD fluorescence (620nm OD/weight)
Spike RBD-Fc
Mouse VEGF antibody

Figure EV5.

Figure EV5. Murine VEGF antibody inhibit spike-induced hyperpermeability and intestinal inflammation.

A The expression of VE-cadherin (VE-cad) and pVE-cad (731) in HUVECs co-cultured with the supernatant from Caco-2 cells that were treated with either Control-Fc, Spike RBD-Fc, or Spike RBD-Fc combined with SCH772984 or Bevacizumab by Western blot. β-actin was used as a reference gene.

B Levels of ERK and pERK in intestinal tissues of mice treated with Control-Fc, Spike RBD-Fc, or Spike RBD-Fc combined with SCH772984 or Bevacizumab by Western blot. GAPDH or β-actin was used as a reference gene. Data are shown from three independent biological experiments.

C ELISA analysis of VEGF concentration in duodenum tissues of mice treated with Spike RBD-Fc or Spike RBD-Fc combined with mouse VEGF antibody. For each group, $n = 4$. *n*, biologically independent samples (mice).

D, E Representative H&E images (D) and the quantitative analysis of inflammation (E) of the intestinal tissues from mouse treated with Spike RBD-Fc or Spike RBD-Fc combined with mouse VEGF antibody. Scale bar, 100 μm. For each group, $n = 5$. *n*, biologically independent samples (mice).

F Evaluation of intestinal permeability in animals treated with Spike RBD-Fc or Spike RBD-Fc combined with mouse VEGF antibody by the Evans Blue dye extravasation assay. For each group, $n = 5$. *n*, biologically independent samples (mice).

Data information: Data are shown as mean ± SD. For (B), *P* values are determined by one-way ANOVA; for (C) and (F), *P* values are determined by Student's *t*-test; ns, not significant.

© 2022 The Authors
