# Supplementary material for: SARS‐CoV‐2 spike spurs intestinal inflammation via VEGF production in enterocytes
Source: EMBO Mol Med. 2022 Apr 19;14(5):e14844. doi: 10.15252/emmm.202114844 (PMC9081906; doi:10.15252/emmm.202114844)
Supplement: Supplementary file 2 — Table EV1 [file EMMM-14-e14844-s004.pdf]

Table EV1. The clinicopathological characteristics of COVID-19 patients with GI symptoms

	Test cohort (n=18)	Validation cohort (n=19)
Mean age	44.8	51.2
Sex		
Male	12 (66.7%)	11 (57.9%)
Female	6 (33.3%)	8 (42.1%)
Symptoms		
Disease classification		
Non-severe	14 (77.8%)	9 (47.4%)
Severe	4 (22.2%)	10 (52.6%)
Diarrhoea	3 (16.7%)	6 (31.6%)
Anorexia	0 (0%)	7 (36.8%)
Nausea	3 (16.7%)	4 (21.1%)
Vomit	1 (5.6%)	3 (15.8%)
Acid reflux	3 (16.7%)	0 (0%)
Epigastric discomfort	4 (22.2%)	7 (36.8%)
Hepatic function impairment		
Total bilirubin (μmol/L; normal range 3.0–24.0) Increased	6 (33.3%)	6 (31.6%)
ALT (U/L; normal range 7–40 in female, 9–50 in male) Increased	6 (33.3%)	8 (42.1%)
AST (U/L; normal rage 13–35 in female, 15–40 in male) Increased	11 (61.1%)	6 (31.6%)
serologic markers of disease severity		
CRP (μg/L; normal range 0.068-8.2)	8 (44.4%)	16 (84.2%)
D-dimers (μg/L; normal range 0-243)	7 (38.9%)	12 (63.2%)
Procalcitonin (ng/L; normal range 0-0.5)	3 (16.7%)	4 (21.1%)
Nucleic acid test results of fecal samples (Positive)	16 (88.9%)	7 (36.8%)

Data are presented as n (%) and % is the percentage in 18 patients (test cohort) or 19 patients (validation cohort)

ALT, alanine aminotransferase; AST, aspartate transaminase (value on initial presentation); CRP, C-reactive protein
