# Supplementary material for: SARS‐CoV‐2 spike spurs intestinal inflammation via VEGF production in enterocytes
Source: EMBO Mol Med. 2022 Apr 19;14(5):e14844. doi: 10.15252/emmm.202114844 (PMC9081906; doi:10.15252/emmm.202114844)
Supplement: Supplementary file 6 — Source Data for Figure 3 [file EMMM-14-e14844-s008.zip › EMM-2021-14844-v3_Figure_3_source_data/EMM-2021-14844-v3_Figure_EV3_source_data.pdf]

Fig.3D

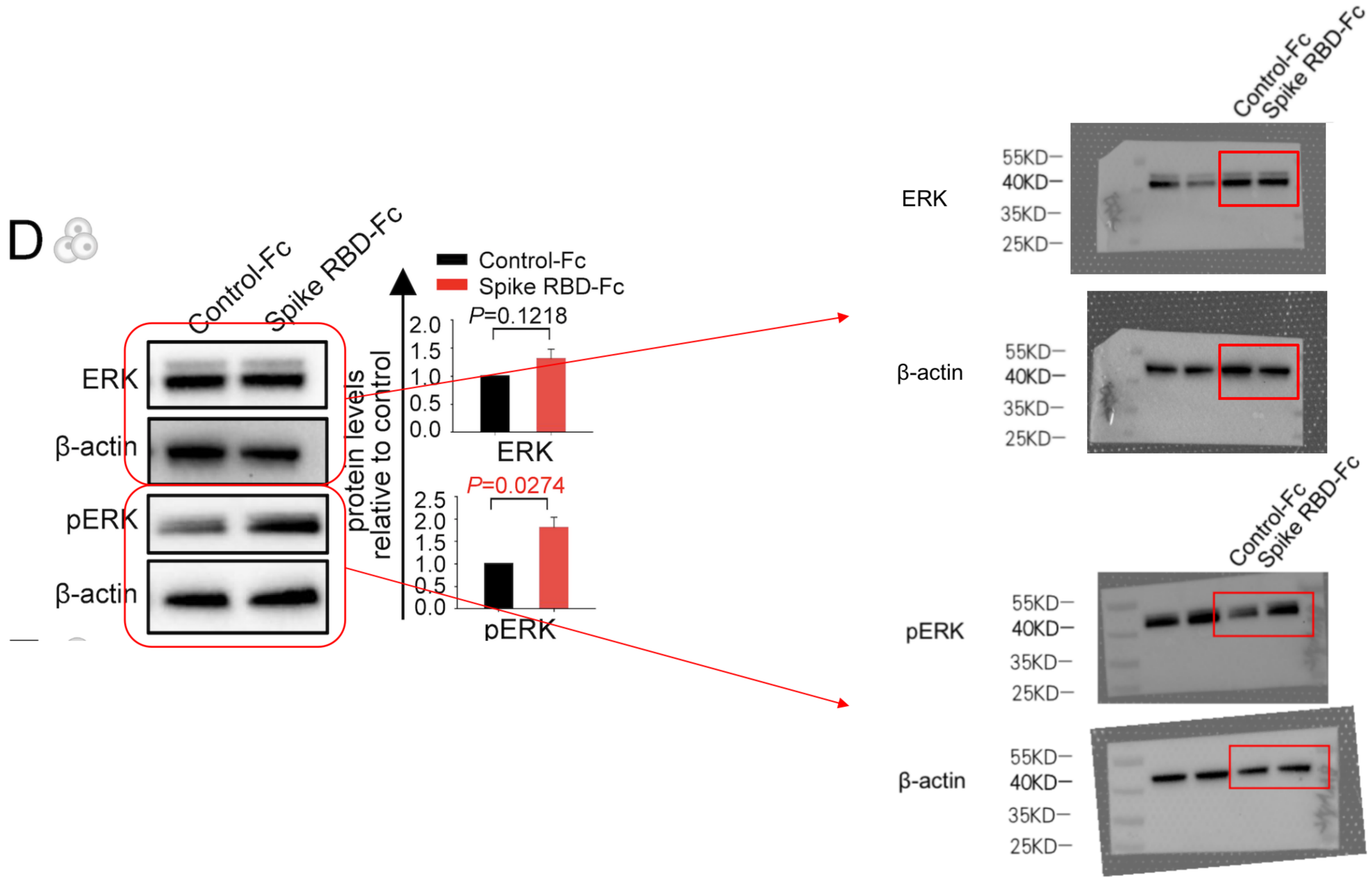

D
Control-Fc
Spike RBD-Fc
ERK
β-actin
pERK
β-actin
protein levels relative to control
P=0.1218
2.0
1.5
1.0
0.5
0.0
ERK
P=0.0274
2.5
2.0
1.5
1.0
0.5
0.0
pERK
55KD
40KD
35KD
25KD

Fig.3F

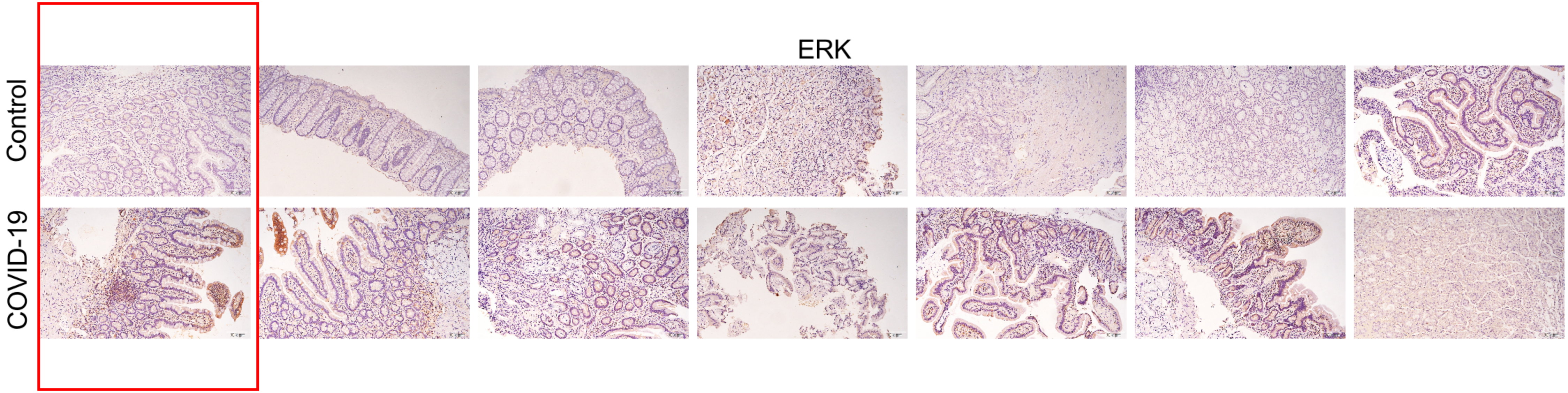

Fig.3G

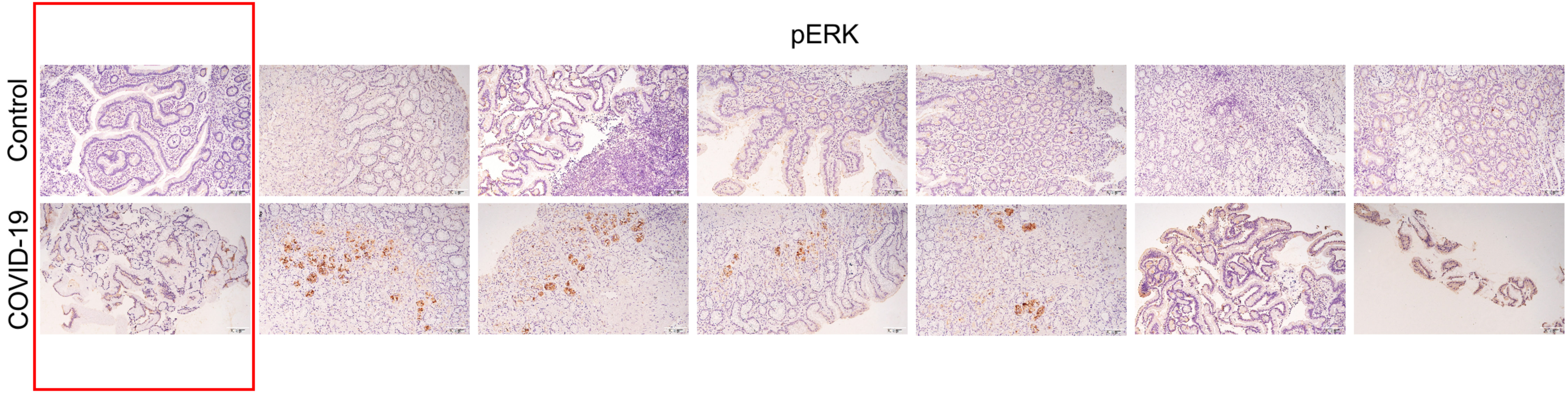

Fig.3H

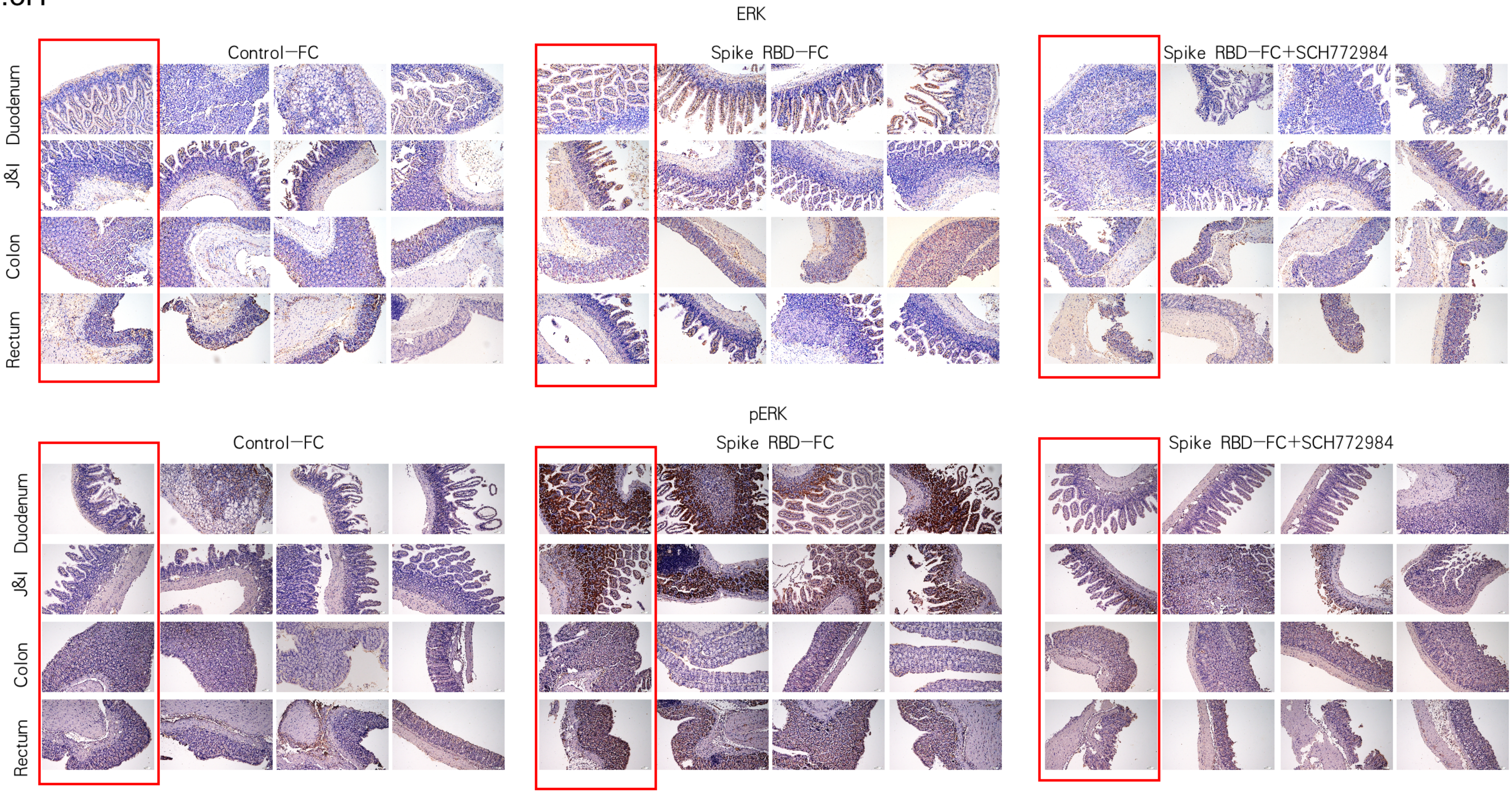
ERK
Control−FC
Spike RBD−FC
Spike RBD−FC+SCH772984
Duodenum
J&I
Colon
Rectum
pERK
Control−FC
Spike RBD−FC
Spike RBD−FC+SCH772984
Duodenum
J&I
Colon
Rectum

Fig.3I

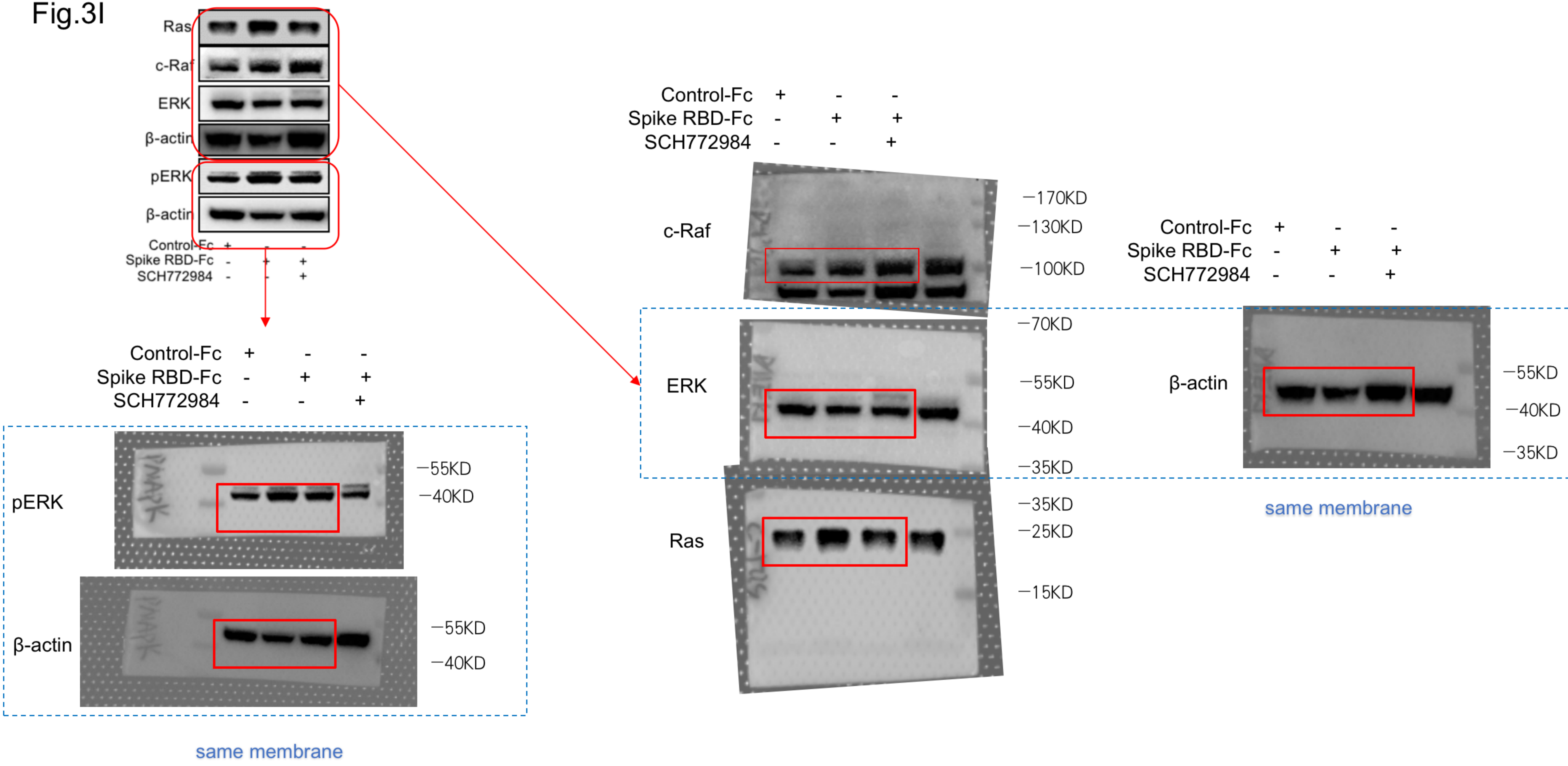

Ras
c-Raf
ERK
β-actin
pERK
β-actin
Control-Fc + - -
Spike RBD-Fc - + +
SCH772984 - - +
Control-Fc + - -
Spike RBD-Fc - + +
SCH772984 - - +
pERK
55KD
40KD
β-actin
55KD
40KD
same membrane
c-Raf
170KD
130KD
100KD
ERK
70KD
55KD
40KD
35KD
Ras
35KD
25KD
15KD
Control-Fc + - -
Spike RBD-Fc - + +
SCH772984 - - +
β-actin
55KD
40KD
35KD
same membrane

Fig.3I

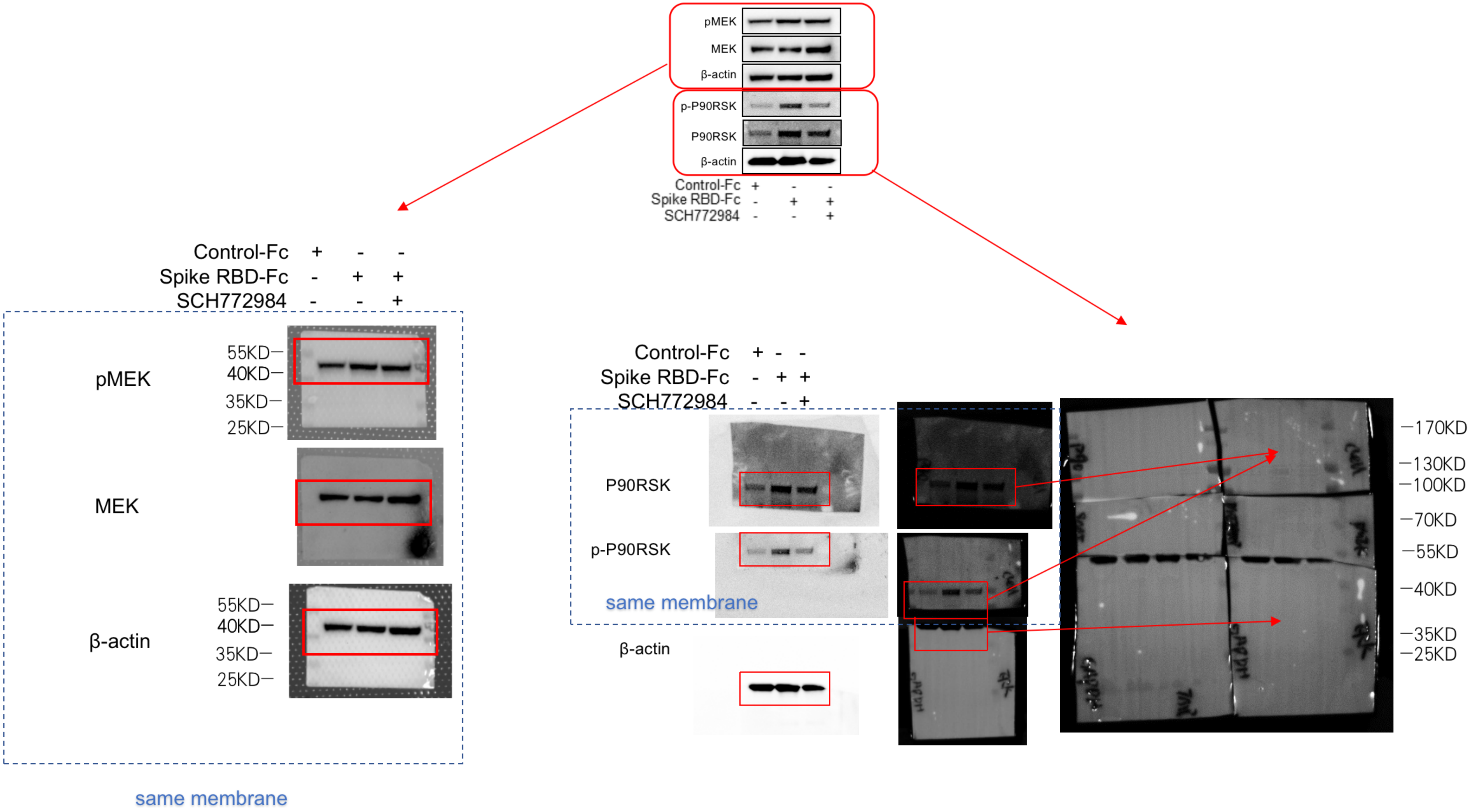

pMEK
MEK
β-actin
p-P90RSK
P90RSK
β-actin
Control-Fc + - -
Spike RBD-Fc - + +
SCH772984 - - +
Control-Fc + - -
Spike RBD-Fc - + +
SCH772984 - - +
pMEK
55KD
40KD
35KD
25KD
MEK
β-actin
55KD
40KD
35KD
25KD
same membrane
Control-Fc + - -
Spike RBD-Fc - + +
SCH772984 - - +
P90RSK
p-P90RSK
same membrane
β-actin
170KD
130KD
100KD
70KD
55KD
40KD
35KD
25KD
