# Supplementary material for: SARS‐CoV‐2 spike spurs intestinal inflammation via VEGF production in enterocytes
Source: EMBO Mol Med. 2022 Apr 19;14(5):e14844. doi: 10.15252/emmm.202114844 (PMC9081906; doi:10.15252/emmm.202114844)
Supplement: Supplementary file 7 — Source Data for Figure 4 [file EMMM-14-e14844-s005.zip › EMM-2021-14844-v2_Figure_4_raw_data/source_data_Fig._4.pdf]

Fig.4B

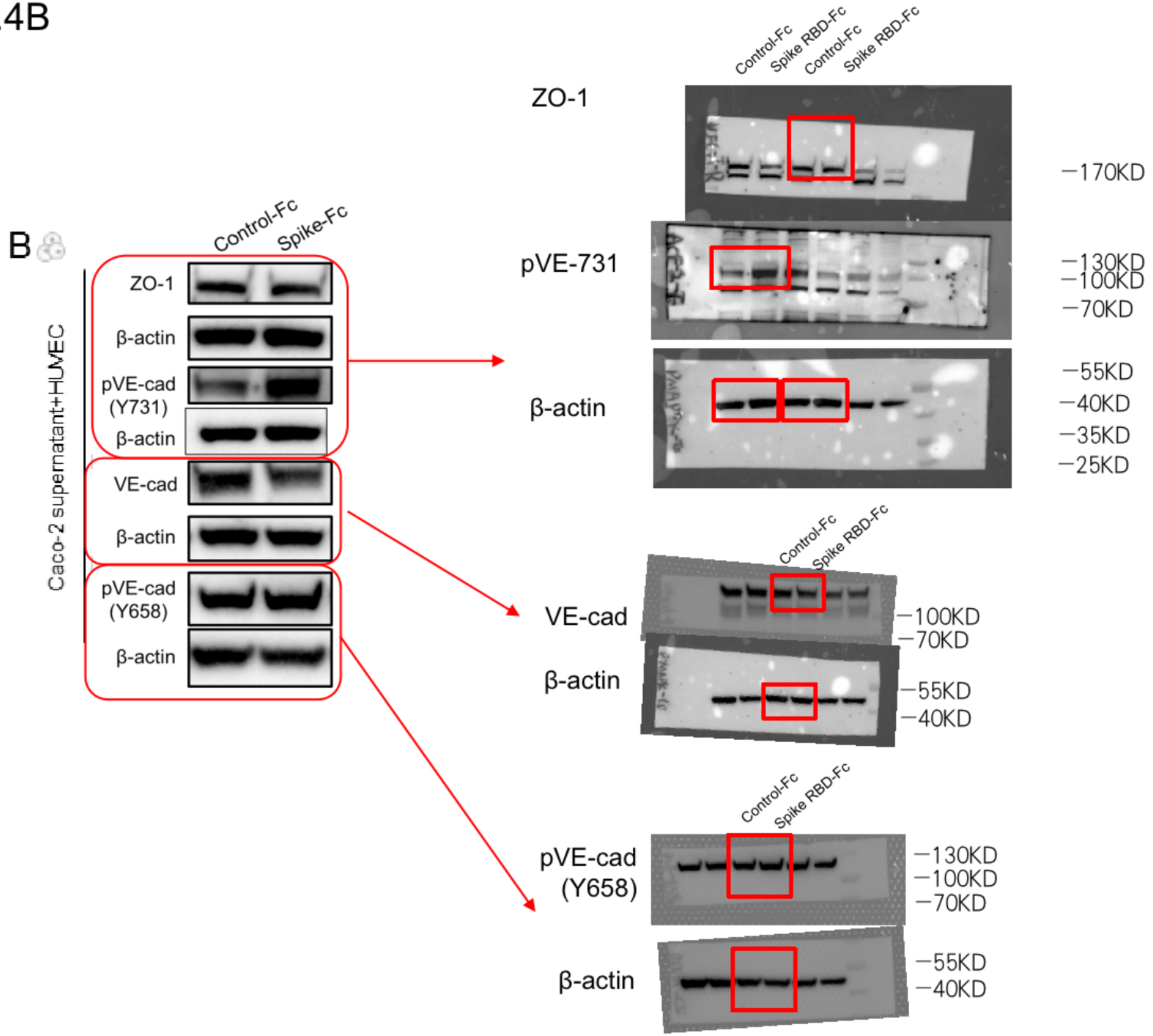
B
Control-Fc
Spike-Fc
Caco-2 supernatant+HUMEC
ZO-1
β-actin
pVE-cad
(Y731)
β-actin
VE-cad
β-actin
pVE-cad
(Y658)
β-actin
Control-Fc
Spike RBD-Fc
Control-Fc
Spike RBD-Fc
ZO-1
170KD
pVE-731
130KD
100KD
70KD
β-actin
55KD
40KD
35KD
25KD
Control-Fc
Spike RBD-Fc
VE-cad
100KD
70KD
β-actin
55KD
40KD
Control-Fc
Spike RBD-Fc
pVE-cad
(Y658)
130KD
100KD
70KD
β-actin
55KD
40KD

Fig.4C

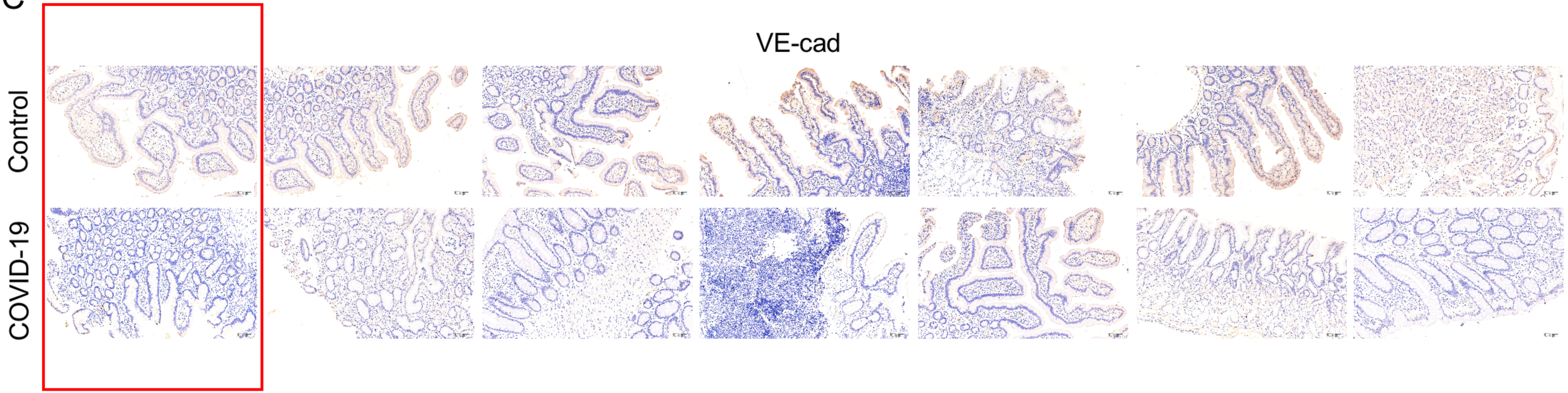

Fig.4D

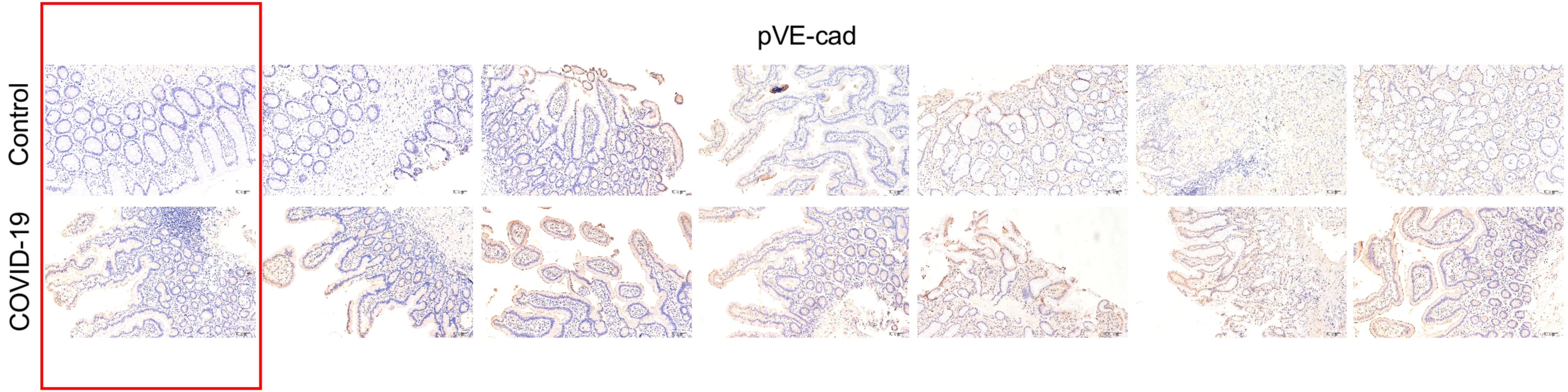

Fig.4G

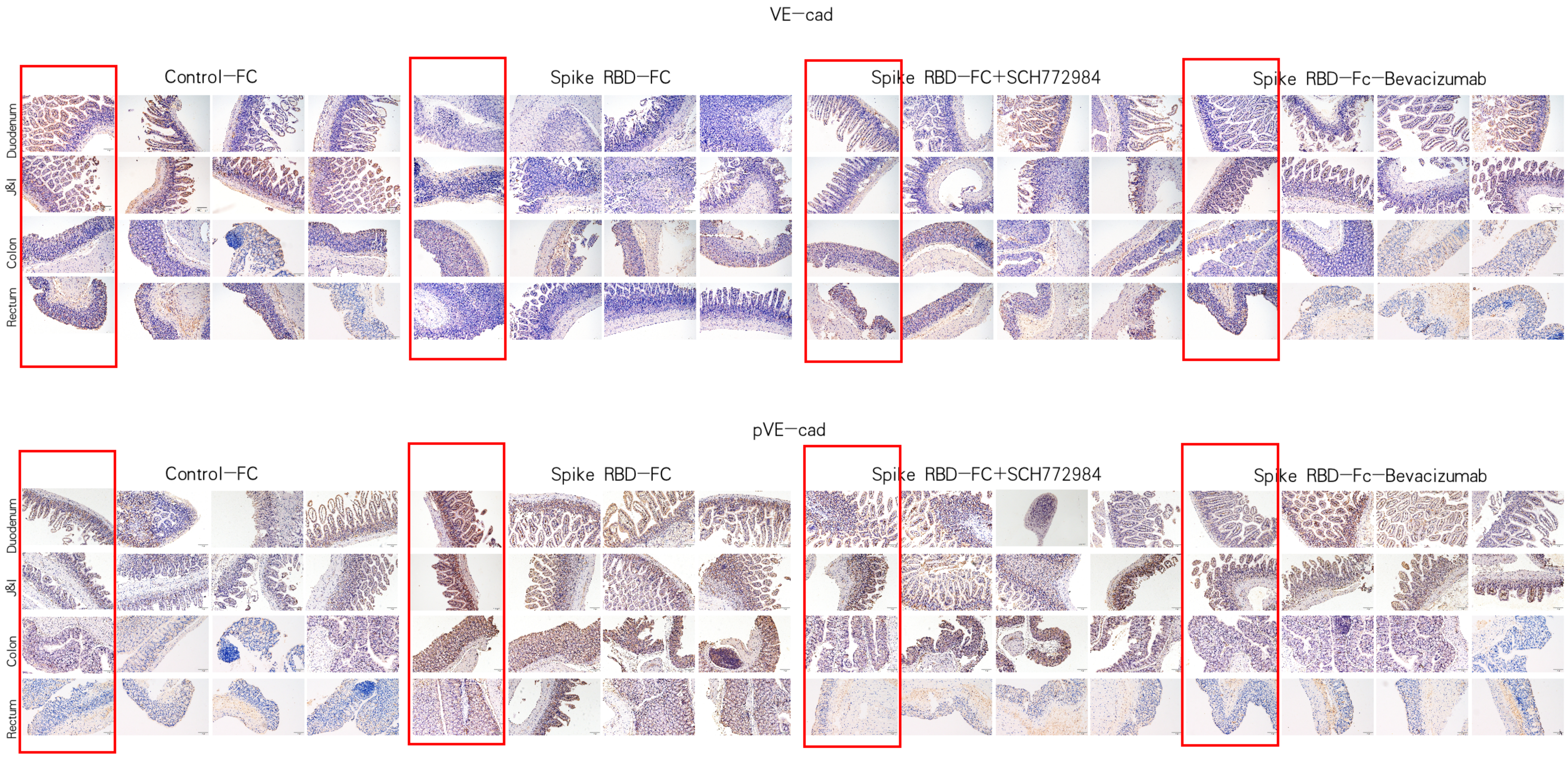
VE−cad
Control−FC
Spike RBD−FC
Spike RBD−FC+SCH772984
Spike RBD−Fc−Bevacizumab
Duodenum
J&I
Colon
Rectum
pVE−cad
Control−FC
Spike RBD−FC
Spike RBD−FC+SCH772984
Spike RBD−Fc−Bevacizumab
Duodenum
J&I
Colon
Rectum
