# Supplementary figures and images for: SARS‐CoV‐2 spike spurs intestinal inflammation via VEGF production in enterocytes
Source: EMBO Mol Med. 2022 Apr 19;14(5):e14844. doi: 10.15252/emmm.202114844 (PMC9081906; doi:10.15252/emmm.202114844)

Fig.5C

Duodenum

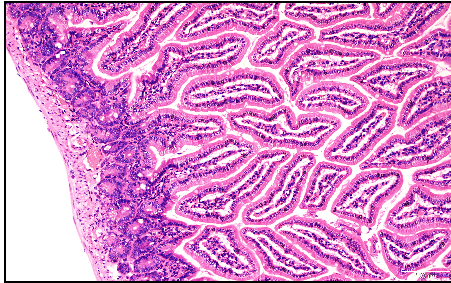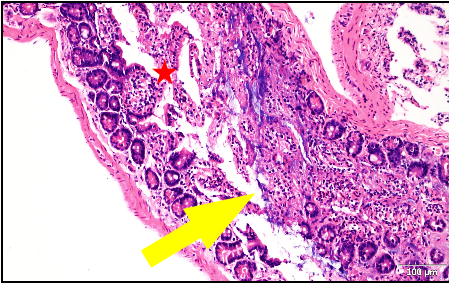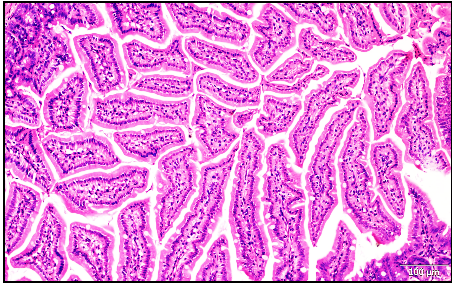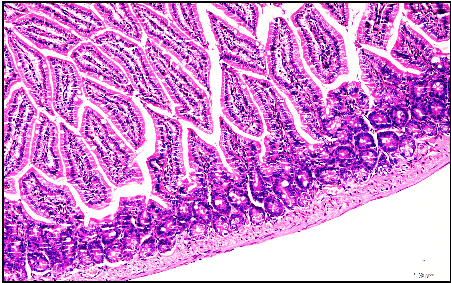

J&I

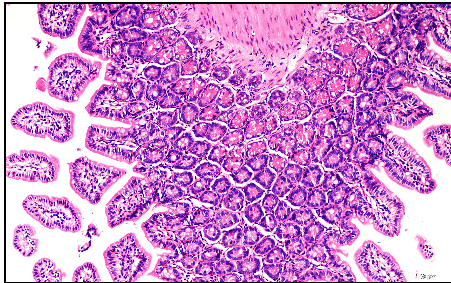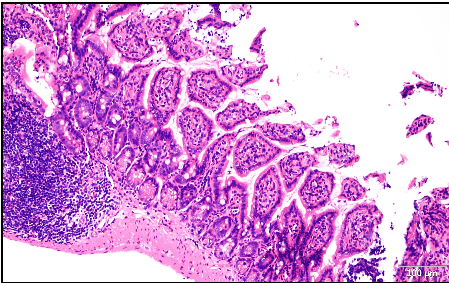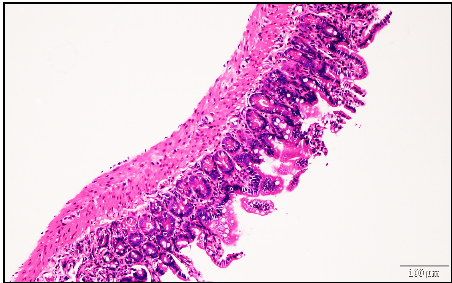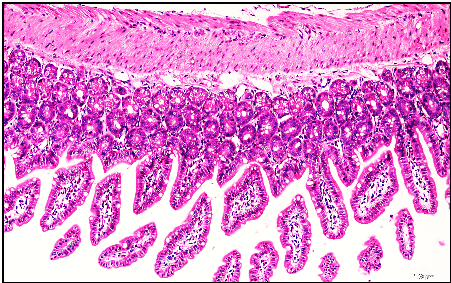

Colon

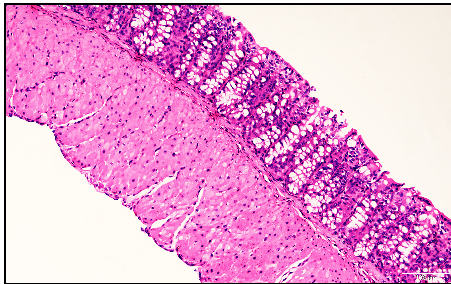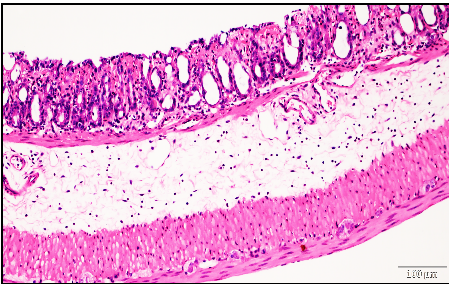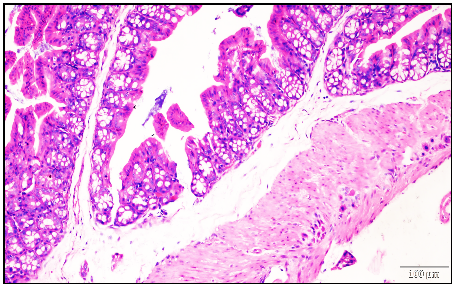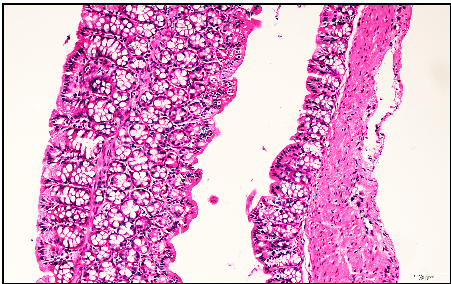

Rectum

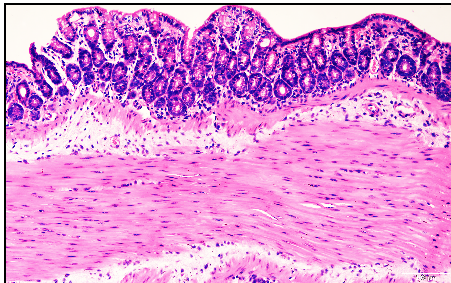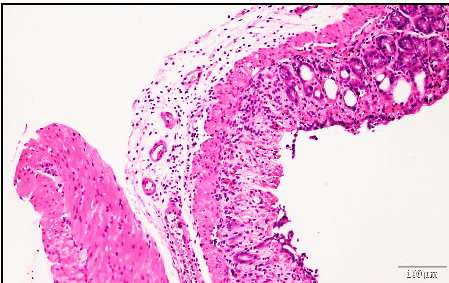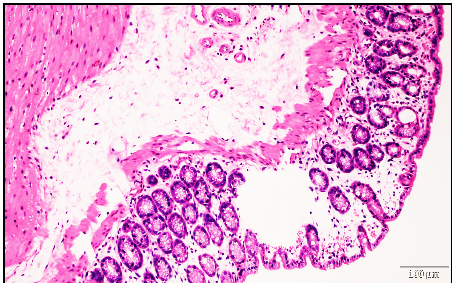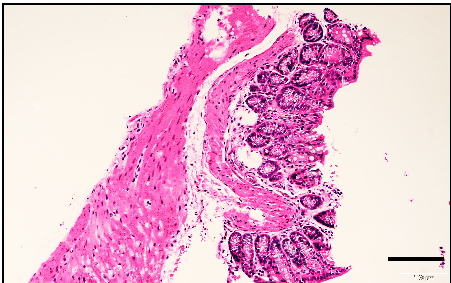

Bevacizumab  
Control-Fc  
Spike RBD-Fc  
SCH772984

+  
-  
-  
-

+  
-  
-  
-

-  
+  
+  
-

-  
+  
-  
+

Supplement: Supplementary file 8 — Source Data for Figure 5 [file EMMM-14-e14844-s006.zip › EMM-2021-14844-v2_Figure_5_raw_data/source_data_Fig._5.pdf]
